# Integrated Transcriptomic and Metabolomic Profiling of Paclobutrazol-Induced Dwarfism in Tomato Epicotyls

**DOI:** 10.3390/plants14213311

**Published:** 2025-10-30

**Authors:** Junqi Wang, Jinzhe Li, Changxin Xiao, Yingbin Qi, Bing Bai, Xia Cao, Xiujie Mao, Chuncheng Wu, Qun Liu, Mingjia Tang, Ning Zhang

**Affiliations:** 1 College of Horticulture Science & Technology, Hebei Normal University of Science & Technology, Qinhuangdao 066004, China; 15841005297@163.com (J.W.); ljz0902022403@hevttc.edu.cn (J.L.); 18285798704@163.com (C.X.); shuaibin678@163.com (Y.Q.); baibing1112@126.com (B.B.); caoxia_xy@163.com (X.C.); maoxiujie@126.com (X.M.); wuchuncheng1979@126.com (C.W.); 2 Hebei Key Laboratory of Horticultural Germplasm Excavation and Innovative Utilization, Qinhuangdao 066600, China; 3 Hebei Higher Institute Application Technology Research and Development Center of Horticultural Plant Biological Breeding, Qinhuangdao 066004, China; 4 Jiangsu Key Laboratory for Conservation and Utilization of Plant Resources, Institute of Botany, Jiangsu Province and Chinese Academy of Sciences, Nanjing 210014, China; 5 National Key Laboratory of Crop Genetics and Germplasm Enhancement, College of Horticulture, Nanjing Agricultural University, Nanjing 210095, China; t2024074@njau.edu.cn

**Keywords:** paclobutrazol, tomato, epicotyl dwarfism, transcriptome, metabolome

## Abstract

Tomato (*Solanum lycopersicum* L.) seedlings are prone to excessive growth in summer, especially severe overgrowth of the embryo axis. Paclobutrazol is a plant growth inhibitor that regulates the balance of hormones in plants and delays their growth. In this study, 200 mg·L^−1^ paclobutrazol was sprayed onto highly homozygous inbred strain DH tomato seedlings at the two-leaf stage, which led to a significant reduction in the length of the epicotyl, an increase in the number of cells, a close cell arrangement, and a reduction in cell size. To study the mechanism by which paclobutrazol dwarfs the epicotyl of tomatoes, we utilized a combined analysis of the transcriptome and metabolome to identify potential candidate genes and regulatory pathways. The results revealed that after paclobutrazol treatment, both the flavonoid and phenylpropanoid biosynthesis pathways were jointly annotated. In addition, plant hormones and sucrose metabolism pathways were also discovered using transcriptome analysis. Xyloglucan endotransglucosylase/hydrolases (*XTHs*), small auxin-up RNAs (*SAURs*) and invertase family-related genes were detected, which can serve as key candidate genes for the subsequent analysis of epicotyl dwarfism in tomato plants. These results provide a framework for understanding the metabolic processes underlying epicotyl dwarfism and a foundation for preventing tomato seedling overgrowth.

## 1. Introduction

To ensure a winter supply of greenhouse tomatoes, seedlings must be cultivated in summer; however, this can lead to excessive embryo axis elongation in tomato plants. In overgrown plants, most photosynthates are allocated to the apical meristem, leaving only a small portion for fruit development, which ultimately reduces yield [1]. Embryo axis elongation may be regulated by multiple factors, including plant hormones, sucrose metabolism, and the phenylpropanoid and flavonoid biosynthetic pathways.

Plant hormones are key regulators of epicotyl elongation. Inhibition of gibberellin (GA) biosynthesis or signaling, for instance, results in shorter epicotyls [2]. *Arabidopsis* mutants with defective GA synthesis exhibit this phenotype—a condition that can be significantly reversed by exogenous GA application [3]. Cytokinin (CTK) concentrations also directly influence epicotyl length, with higher CTK levels leading to shorter epicotyls. Furthermore, complex hormonal interactions are involved; CTKs interact with ethylene (ETH) and GA, and ETH can indirectly regulate growth by modulating CTK signaling pathways [4]. This evidence collectively underscores the critical role of phytohormones in epicotyl dwarfing. As a plant growth inhibitor, paclobutrazol (PBZ) delays growth by regulating this hormonal balance. By suppressing vegetative growth, PBZ can ultimately enhance crop yield. The plant growth retardant PBZ suppresses elongation primarily via its triple-blockade of the terpenoid pathway, thereby disrupting GA biosynthesis [5]. The associated reduction in endogenous GA activity induces radial cell expansion, which is manifested as increased cortical thickness, vascular bundle size, and pith diameter, resulting in overall thicker stems [6]. Supporting this, studies of tomato plants confirm a significant reduction in internode length following PBZ treatment, with the effect being concentration-dependent (400 ppm > 200 ppm) [7].

During the hormone-induced promotion of embryo axis elongation, not only is cellular architecture altered but the energetic status of the cell is also profoundly impacted. In this process, the metabolism, transport, and utilization of sugars serve as a critical link. Sucrose acts as a messenger that moves from the cotyledon to the hypocotyl, thereby regulating hypocotyl elongation [8]. Chen et al. (2024) found that the starch and sucrose pathways regulate early seedling development in rapeseed via ABA signaling [9]. GA2ox8c inhibits epicotyl and hypocotyl elongation, and MYB33-SWEET11/21 targets GA2ox8c to regulate hypocotyl elongation via long-distance sucrose transport in soybean plants [10]. Cell wall invertase (CWIN) in the coleoptiles and mesocotyls also affects sucrose unloading in etiolated maize seedlings [11]. These studies indicate that sugars play an important role in embryonic axis development.

Complex interactions exist between plant hormone signaling and phenylpropanoid biosynthesis pathway. Hormones affect plant growth and development by regulating the expression of key genes involved in the phenylpropanoid biosynthesis pathway, which affects the growth phenotype of plants by regulating auxin (Aux) signaling [12]. Alterations in the phenylpropanoid biosynthetic pathway may regulate plant growth by influencing cell wall elasticity and lignin synthesis [13]. Flavonoids and their derivatives, such as flavonols, are secondary metabolites of phenylpropane that play important roles in establishing embryo axis polarity [14,15]. Flavonoids can regulate reactive oxygen species homeostasis and the transport of Aux, thereby influencing the growth direction and morphology of the embryo axis [16]. These previous results indicate that flavonoids affect the growth of the embryo axis by regulating the Aux signaling pathway.

Embryo axis growth and development are regulated at multiple levels; however, a full understanding of this regulatory blueprint ultimately relies on the global data provided by omics approaches. Omics technology can be used to determine the roles and interrelationships of key genes, regulatory factors, and metabolites in complex biological networks, such as signal transduction and metabolic regulation. Ohtaka et al. (2020) revealed that day and night temperatures affected stem elongation in tomato seedlings via regulation of GA and Aux synthesis based on transcriptome and phytohormone profiles. Transcriptomic analysis also revealed that GA3 treatment elicits the expression of the cell-wall-associated gene XYLOGLUCAN ENDOTRANS-GLUCOSYLASE/HYDROLASE 1, which increases tomato plant height [17], and miR394 represses the transcription of PACLOBUTRAZOL RESISTANCE1/5/6 to regulate hypocotyl elongation in *Arabidopsis thaliana* [18]. Using metabolome and transcriptome analyses, Chen et al. (2024) further revealed the changes in rapeseed during early seedling development [9]. Zhang et al. (2022) also revealed the molecular basis for relieving epicotyl dormancy in *Polygonatum cyrtonema* Hua seeds under exogenous 6-benzylaminopurine (6-BA) treatment through transcriptomic and metabolomic analyses [19]. These studies indicate that, through transcriptomics and metabolomics, the regulatory networks of genes and metabolites that drive plant development can be elucidated.

Previous studies have shown that spraying 200 mg·L^−1^ PBZ significantly reduced the length of the tomato epicotyls; however, the specific underlying regulated pathways remain unclear [20]. Therefore, in this study, to identify the regulatory pathways underlying tomato epicotyl dwarfism, we conducted transcriptomic and metabolomic analysis on DH tomato seedling epicotyls at 0, 5, 15, and 25 d after foliar application of 200 mg·L^−1^ PBZ at the two-leaf stage. Significantly enriched genes or metabolites related to plant hormone signal transduction, sucrose metabolism, and phenylpropanoid and flavonoid biosynthesis were also assessed. Our results lay the groundwork for a detailed elucidation of the molecular mechanism controlling PBZ-induced epicotyl dwarfing in tomatoes.

## 2. Results

### 2.1. PBZ-Dwarfed Epicotyls in Tomato Seedlings

**Figure 1:**
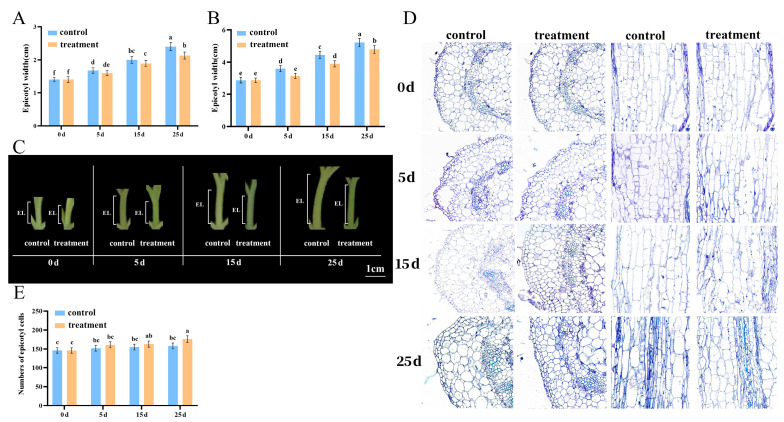
Characteristics of the epicotyls on tomatoes seedlings after paclobutrazol treatment. (**A**) Epicotyl (EL) length. (**B**) Epicotyl width. (**C**) Phenotypic characteristics of epicotyls. (**D**) Cytological characteristics of epicotyls. (**E**) Numbers of epicotyl cells. Data represent the means of three biological replicates (±SD). Different letters indicate significant differences (*p* ≤ 0.05) according to Duncan’s test.

To illustrate the dwarfing effect of PBZ on tomato epicotyls, we analyzed their phenotypic characteristics. We found that compared to that at 0 d, the lengths of epicotyls under the control at 5 d, 15 d, and 25 d increased by 16.7%, 19%, and 20%, respectively, compared to 16.7%, 18%, and 12.5%, respectively, under the PBZ treatment, with the greatest difference at 25 d (Figure 1A–C). Compared to the control, epicotyl thickness was significantly different at 5 d after PBZ treatment than control (Figure 1B,C). Differences in the lengths of the tomato epicotyls are usually due to changes in the number or size of cells. Therefore, cytological observations were conducted, which showed that the cell volumes of PBZ-treated epicotyls decreased, the number of cells increased, and the cell arrangement was tighter than that of the control as the number of days after treatment increased (Figure 1D,E).

### 2.2. Transcriptomic Analysis of Epicotyls Dwarfed by PBZ Treatment

**Figure 2:**
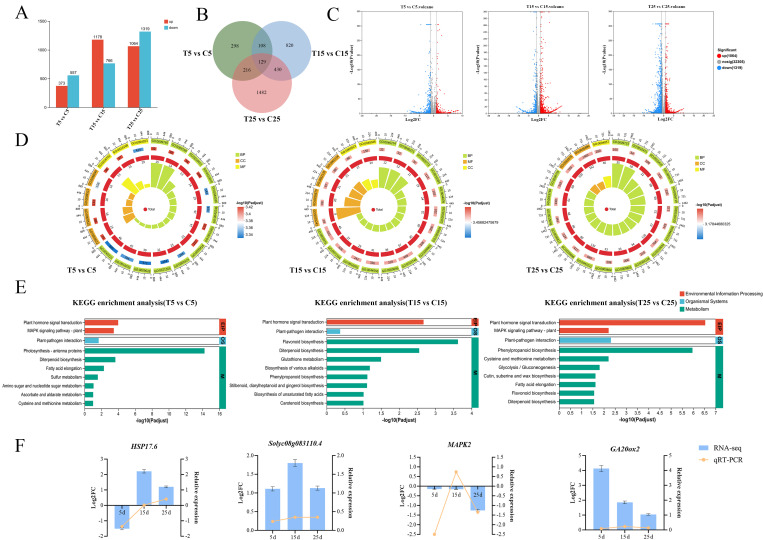
Transcriptome analysis and fold-changes of DEGs. (**A**) Numbers of upregulated and downregulated DEGs under the PBZ treatment compared with those under the control treatment (fold-change ≥ 2.0, *p* ≤ 0.05). The numbers on the bar charts represent the number of genes expressed in each sample. (**B**) Venn diagram showing the number of DEGs after PBZ treatment compared with the control (fold-change ≥ 2.0, *p* ≤ 0.05). Numerals inside parentheses indicate the number of genes expressed in each sample. (**C**) Comparison of DEGs by volcano plots. (**D**) GO annotation analysis on different days of PBZ treatment. (**E**) KEGG enrichment analysis on different days of PBZ treatment. (**F**) DEG expression was determined and verified on different days of PBZ treatment. The left *Y*-axis represents the baseline level of DEGs in the transcriptome and the right *Y*-axis represents the relative expression of DEGs, as determined by RT-qPCR. Data represent the means of three biological replicates (±SD).

To identify the potential regulatory genes of the epicotyls of tomatoes dwarfed by PBZ treatment, we performed transcriptome analysis of DH seedlings treated with PBZ for different numbers of days. In total, 136.85 Gb of clean data were obtained, and each clean data were reaching over 6.03 Gb. The base percentage of Q30 was above 95.96%. The clean reads of each sample were sequentially aligned with the designated reference genome, with alignment rates ranging from 96.9% to 98.2% (Table S1). In total, 30,990 differentially expressed genes (DEGs) were identified, of which 15,315 and 15,675 were upregulated and downregulated, respectively. The number of DEGs screened: 373, 1178, 1064 upregulated and 557, 766, 1319 downregulated genes, respectively, at three treatment groups (Figure 2A). A total of 129 DEGs were detected, and the distribution of upregulated and downregulated genes was relatively uniform at three treatment groups (Figure 2B,C). GO annotation analysis revealed enriched metabolic and cellular processes at 5, 15, and 25 d (Figure 2D). KEGG enrichment analysis revealed plant hormone signal transduction and flavonoid biosynthesis and their potential involvement in epicotyl dwarfism (Figure 2E). To validate the transcriptome results, four genes annotated in GenBank were selected for qRT-PCR. Except for *MAPK2*, the regulatory trends of the other three genes were consistent, confirming that the transcriptome results were reliable and could be used for further analyses (Figure 2F). Differentially expressed transcription factors (TFs) obtained from the treatment and control were statistically analyzed. Of these, the genes encoding ERF, MYB, WRKY, NAC, and bHLH families were the most differentially expressed TFs. The ERFs showed the highest number of significantly expressed genes in the treatment and control (Table S2).

**Figure 3:**
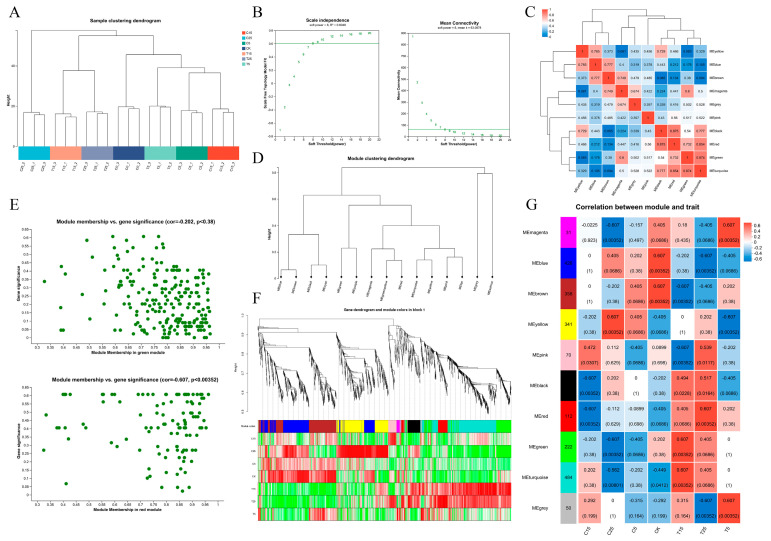
Transcriptome weighted gene co-expression network analysis. (**A**) DEG sample clustering analysis diagram. (**B**) Scale-free fit curve and average connectivity curve. (**C**) Module correlation clustering heatmap. Different colors represent different modules. (**D**) Module clustering tree diagram. (**E**) MM-GS analysis. (**F**) Clustering of gene–phenotype correlations. (**G**) Module–phenotype correlation clustering.

Weighted gene co-expression network analysis (WGCNA) was used to conduct a co-expression network analysis of unigenes in the different treatments and to explore the relationships and networks among various genes. After eliminating the outlier samples, a scale-free fit curve and an average connectivity analysis were conducted. As the scale-free fit curve was in the smooth area, the power-exponential weighted β value, that is, the soft threshold, was set at 8 (Figure 3A,B). Expression-pattern-related genes were grouped into the same module, and ten modules were divided using a clustering tree constructed based on the correlation coefficients between the genes (Figure 3C). Modules with strong correlations were clustered using a module-clustering tree diagram, and modules of the same color were grouped into one category (Figure 3D). Module membership–gene significance (MM–GS) analysis was used to select the red and green modules with high GS and module eigengene-based connectivity (kME) values (Figure 3E). There were significant differences, and genes showed high expression levels between the treatment and control in the red, green, and blue modules based on a comparison of the correlations between modules, genes, and phenotypes (Figure 3F,G).

**Figure 4:**
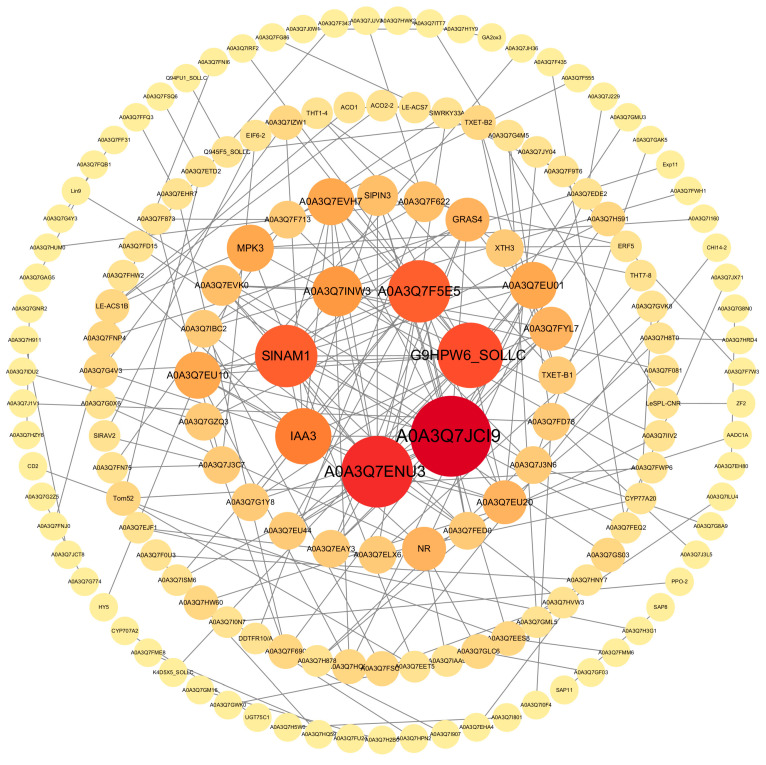
Protein–protein interaction network analysis. Different bubble colors and sizes represent proteins that play important roles in the interaction networks, with those represented by deep colors and large bubbles playing a more important role in network regulation.

**Figure 5:**
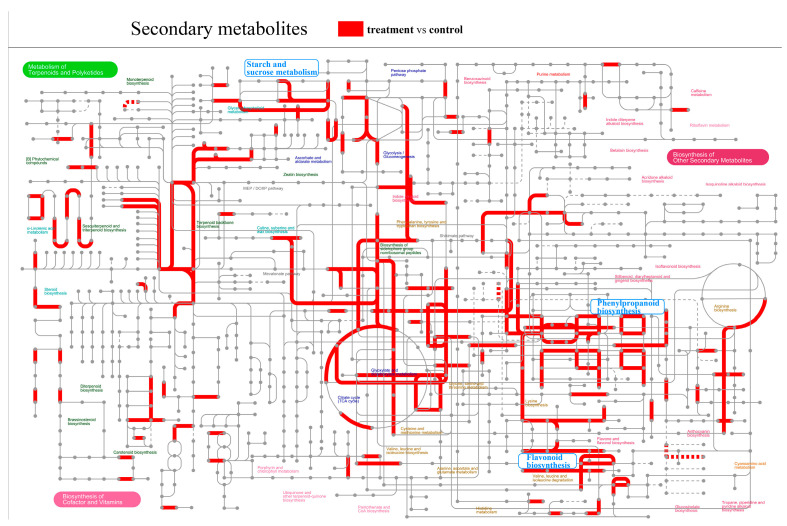
iPath metabolic pathway map. Nodes represent different compounds and edges represent different enzymatic reactions. Red shows all pathways where the differential metabolites of the treatment and control groups are located.

We selected the blue module, which was rich in genes and showed significant differences in the protein–protein interaction network analysis. Proteins such as IAA3, XTH3, Lin9, Exp11, and HY5 in the network are mainly involved in epicotyl growth and development (Figure 4). Statistical analysis of the secondary metabolites in the treatment and control gene sets revealed that the phenylpropanoid biosynthesis pathway, flavonoid biosynthesis, and starch and sucrose metabolism all play roles in epicotyl growth and development (Figure 5).

### 2.3. Effect of Plant Hormones on Epicotyl Dwarfing

**Figure 6:**
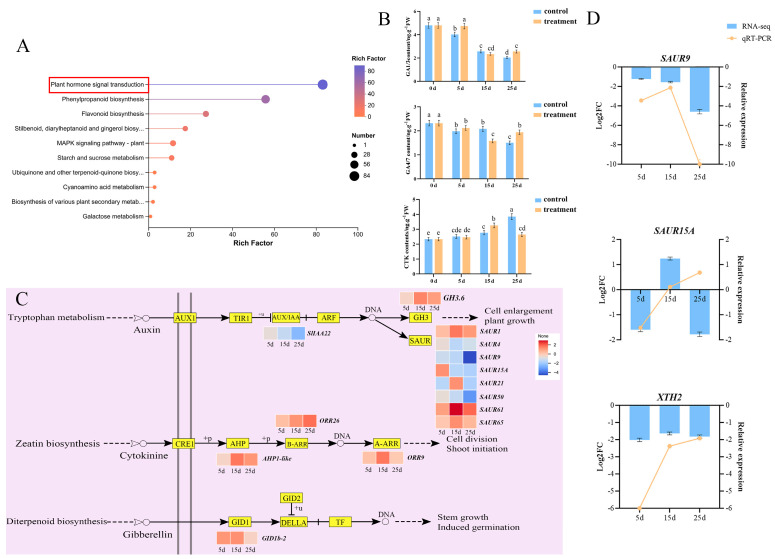
Plant hormones involved in the regulation of epicotyl dwarfing. (**A**) KEGG enrichment analysis of the signal transduction pathway of plant hormones. (**B**) GA and CTK contents on different days of PBZ treatments. Different letters indicate significant differences (*p* ≤ 0.05) according to Duncan’s test. (**C**) Regulatory patterns and DEG changes for plant hormone signal transduction pathways. (**D**) DEG expression was identified and verified on different days of PBZ treatment. The left *Y*-axis represents the basic level of DEGs in the transcriptome, and the right *Y*-axis represents the relative expression of DEGs, as determined using RT-qPCR. Data represent the means of three biological replicates (±SD).

Plant hormones are key factors that affect epicotyl development. KEGG analysis revealed that plant hormone signal transduction was highly enriched (Figure 6A). GA and CTK contents were determined on different treatment days; GA content was elevated at 5 d and 25 d and lower at 15 d after spraying with PBZ, whereas CTK content showed the opposite trend. The trends for GA1/3, and GA4/7 were consistent (Figure 6B). GA and CTK regulatory pathway genes were upregulated, and the expression patterns of Aux regulatory pathway genes were indeterminate (Figure 6C). Thirteen genes were detected, and the expression of three of these was determined by comparing the changes in the expression levels of genes and plant hormones in the KEGG pathway, including two *SAURs*, and *XTH2*. The expression of *SAUR9* and *XTH2* was found to be downregulated based on RT-qPCR, which was consistent with the transcriptome results (Figure 6D). These results indicate that plant hormones are involved in the regulation of epicotyl dwarfing.

### 2.4. Effect of Sucrose Metabolism on Epicotyl Dwarfing

**Figure 7:**
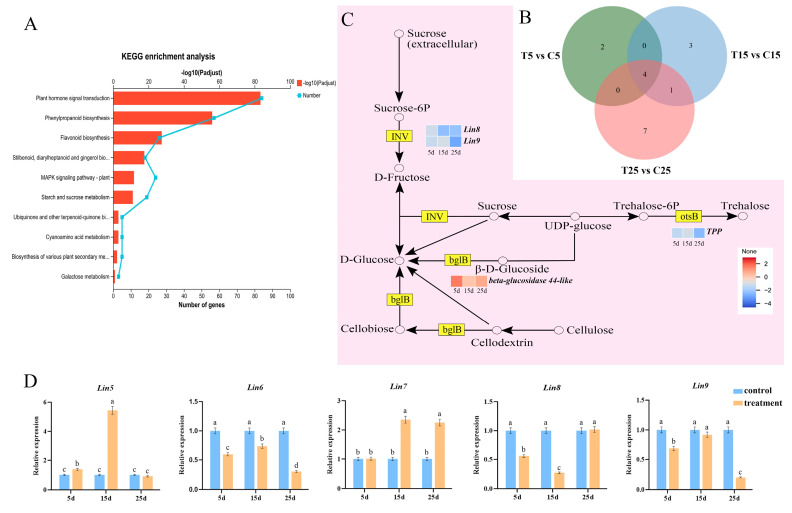
Sucrose metabolism is involved in the regulation of epicotyl dwarfing. (**A**) KEGG enrichment analysis of sucrose metabolism. (**B**) DEG analysis of sucrose metabolism on different treatment days. (**C**) Regulatory patterns and DEG changes in the sucrose metabolism pathway. (**D**) DEG expression was determined and verified on different days of PBZ treatment. The *Y*-axis represents the relative expression level of *Lin5*, *Lin6*, *Lin7*, *Lin8*, and *Lin9* by RT-qPCR. Data represent the means of three biological replicates (±SD). Different letters indicate significant differences (*p* ≤ 0.05) according to Duncan’s test.

KEGG analysis revealed that 19 DEGs were enriched in starch and sucrose metabolisms (Figure 7A). Three DEGs were co-enriched in the groups of data obtained at three time points, whereas one DEG was co-enriched at 15 and 25 d (Figure 7B). The cell wall invertases *Lin8*(*Solyc10g083300.2*) and *Lin9*(*Solyc08g079080.6*), which may inhibit sucrose decomposition, were found to be downregulated based on the transcriptome analysis (Figure 7C). To illustrate the effect of invertase on epicotyl development, the expressions of *Lin5*, *Lin6*, *Lin7*, *Lin8* and *Lin9* were analyzed. Compared with control, the expression of *Lin6*, *Lin8*, and *Lin9* was downregulated after PBZ treatment, whereas *Lin7* expression was upregulated 2.35- and 2.25-fold at 15 and 25 d, respectively, after PBZ treatment. *Lin5* was upregulated at both 5 and 15 d, and compared with control, *Lin5* expression increased 5.44-fold at 15 d after PBZ treatment (Figure 7D).

### 2.5. Effect of the Phenylpropanoid and Flavonoid Biosynthesis Pathway on Epicotyl Dwarfing

**Figure 8:**
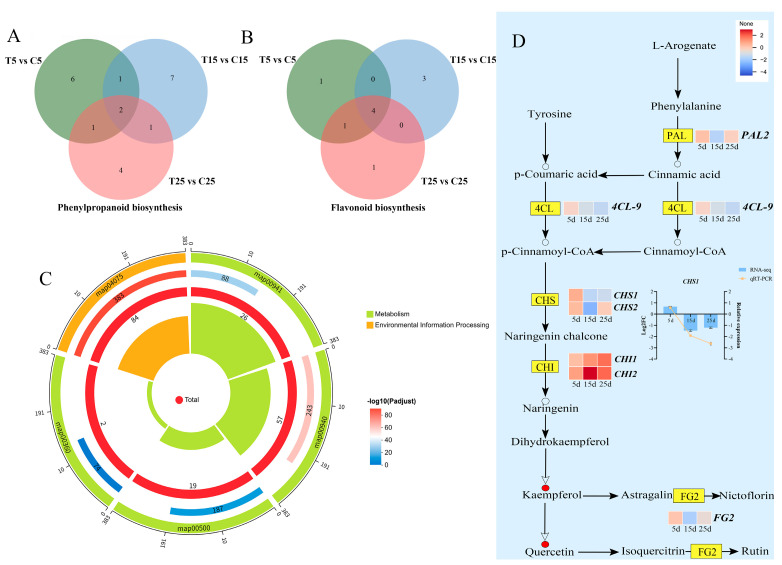
Phenylpropanoid biosynthesis and flavonoid biosynthesis are involved in the regulation of epicotyl dwarfing. (**A**) DEG number analysis of the phenylpropanoid biosynthesis pathway on different treatment days. (**B**) DEG number analysis of the flavonoid biosynthesis pathway on different treatment days. (**C**) KEGG enrichment analysis of phenylpropanoid biosynthesis and flavonoid biosynthesis. (**D**) Regulatory patterns and DEG changes for the phenylpropanoid biosynthesis pathways.

Phenylpropane can promote cell division and elongation and, therefore, affect epicotyl length, with two DEGs identified among the three treatment groups (Figure 8A). Flavonoids, which are secondary metabolites of the phenylpropanoid pathway, also affect plant growth and development, with two DEGs among the three treatment groups (Figure 8B). The multidimensional enrichment circle diagram showed that the phenylpropanoid biosynthesis and flavonoid biosynthetic pathways were enriched with 57 and 26 DEGs, respectively (Figure 8C). The regulatory pattern diagram shows the differential expression of phenylalanine (*PAL*) and 4-cinnamoyl-CoA (*4CL*) in the phenylpropanoid biosynthesis pathway, which may be involved in regulating epicotyl development. The differential expressions of chalcone synthase (*CHS*) and chalcone isomerase (CHI) were identified in the flavonoid biosynthesis pathway, which may be involved in regulating epicotyl development. Additionally, the same regulatory pathways as those in the transcriptome were discovered in the metabolome, namely the phenylpropanoid and flavonoid biosynthesis pathways. Two differential metabolites involved in the flavonoid biosynthesis pathway, kaempferol and quercetin, showed upregulated expression at 5 and 15 d after PBZ treatment (Figure 8D).

### 2.6. Metabolomic Analysis of Epicotyls Dwarfed by PBZ Treatment

**Figure 9:**
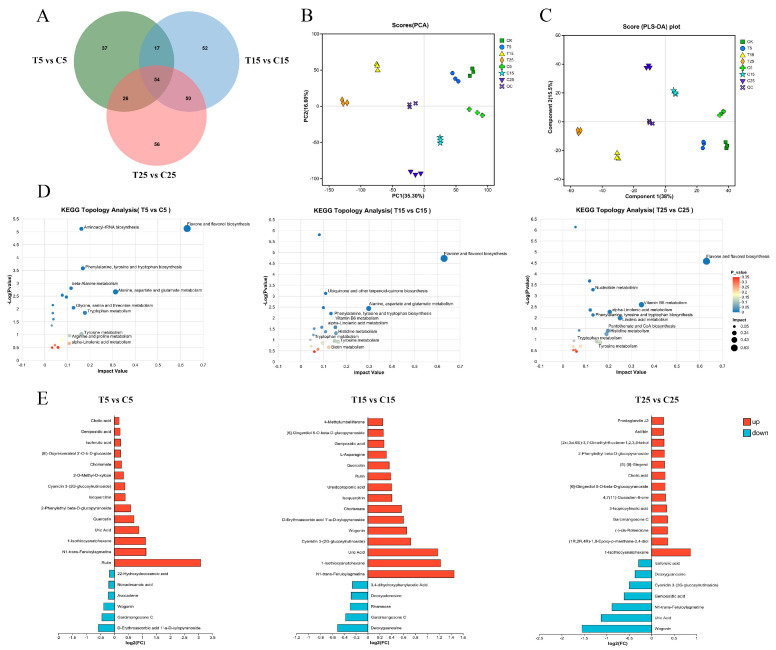
Metabolome analysis on different days after PBZ treatment. (**A**) Numbers of DAMs on different days after PBZ treatment. (**B**) Principal component analysis (PCA) plots and PLS-DA score plots on different days after PBZ treatment. (**C**) KEGG topology analysis on different days after PBZ treatment. (**D**) Column chart of fold differences between groups. (**E**) Statistical analysis of DAMs between control and treatment groups on different days after PBZ treatment. The *X*-axis and *Y*-axis represent log_2_FC and metabolites, respectively. Red and blue columns represent up- and down-regulation, respectively, and the length of the columns represents the regulated multiple.

To verify the regulatory pathways involved in epicotyl dwarfing in tomatoes identified by transcriptome analysis, a metabolome analysis was conducted. A total of 2732 differentially accumulated metabolites (DAMs) after PBZ treatment were identified, of which 1990 were upregulated and 742 were downregulated. The most quantified DAMs were obtained 25 d after PBZ treatment, totaling 186, including 148 upregulated and 38 downregulated DAMs. The least number of DAMs (134; 69 upregulated and 65 downregulated) were quantified at 5 d (Table S3). A total of 54 DAMs were jointly annotated at 5, 15, and 25 d, and included L-tyrosine, indoleacrylic acid, valtrate, L-tryptophan, wogonin, and testosterone (Figure 9A). The quality control samples were well grouped, indicating that the bioanalysis and data quality were good according to the PCA. This suggests that the separation between the groups was caused by differential variables rather than by differences during the analysis process (Figure 9B). The degree of separation of the samples in each group was relatively high, indicating that there were significant differences between the treatment and control groups based on PLS–DA (Figure 9C). The flavone and flavonol biosynthesis pathways and the phenylalanine, tyrosine, and tryptophan biosynthesis pathways were identified at 5, 15, and 25 d after PBZ treatment based on the KEGG topology analysis. The impact value of the flavone and flavonol biosynthesis pathways was the greatest, which may play an important role in PBZ dwarfing the epicotyl (Figure 9D). Based on statistical analysis of the differential metabolites among the three treatments, flavonoid substances, such as wogonin and cyanidin 3-(2G-glucosylrutinoside), and terpenoid substances, such as geniposidic acid, were detected. Quercetin, isoquercetin, and rutin were significantly different between the comparison treatments at 5 and 15 d. These metabolites were involved in the phenylpropanoid and flavonoid biosynthesis pathways, consistent with the results of the transcriptome analysis (Figure 9E).

## 3. Discussion

The length of the embryonic axis of tomatoes is an important factor affecting plant height at the seedling stage. The application of PBZ can slow the growth rate of the epicotyl and dwarf plants, thereby preventing the overgrowth of seedlings [20].

During plant growth and development, the size and division rate of cells play crucial roles in determining the length of the epicotyl at the seedling stage. They are regulated by plant hormone signals, such as IAAs, GAs, and CTKs, and play a key role in plant height [17,21,22]. Previous studies have primarily focused on the regulation of hypocotyl elongation by plant hormones; however, only a few studies have addressed the regulation of epicotyl elongation [20]. In the present study, epicotyl cells became smaller and more numerous after 15 d of PBZ treatment (Figure 1D), the GA content decreased, and the CTK content increased up to 15 d of PBZ treatment (Figure 6B). However, there was little difference in the cell structure after 5 and 15 d of PBZ treatment, with the strongest dwarfing effect observed at 25 d. Meanwhile, CTK-regulated pathway genes were upregulated after 15 d of PBZ treatment (Figure 6C). Although the length of the epicotyl was dwarfed, the difference was not significant at 5 and 15 d after PBZ treatment, indicating that PBZ treatment inhibited GA synthesis, leading to the dwarfing of the epicotyl. It is precisely because of the decrease in GA content that the content of CTKs increases to maintain the normal division of cells. Surprisingly, the content of active GA suddenly increased and the content of CTK decreased at 25 d after PBZ treatment. This might be because tomato epicotyl perceived the state of GA deficiency and triggered a compensatory mechanism to maintain its own GA demand. In addition, Liu et al. (2022) found that IAA content was increased by 200 mg·L^−1^ PBZ treatment [20]. In this study, *SAUR9* was downregulated, which is consistent with the cell phenotype.

XTHs are a class of cell wall-modifying proteins that also have the capacity to loosen cell walls and promote cell elongation [23]. Many studies have found that *XTH* either stimulates growth or does not affect plant hypocotyls [24,25,26,27]. The overexpression of *AtXTH18*, *AtXTH19*, and *AtXTH20* stimulates growth and cell wall mechanics in etiolated *A. thaliana* hypocotyls [23]. Luo et al. (2024) found that *XTH19* increases the height of tomato plants and affects epicotyl development [28]. In this study, *XTH2* expression was downregulated by PBZ treatment, indicating that it did not promote cell elongation and could be a consequence of growth inhibition (Figure 6D). This effect was also observed in radish, with PBZ blocking GA-induced etiolation by decreasing *EXP*, *XTH* and *YUC* expression [29]. Furthermore, the expression of XTHs is regulated by multiple plant hormones; high concentrations of Aux inhibit cell expansion by suppressing XTH expression [30], while low concentrations of Aux and GA relieve *XTH* inhibition through DELLA protein degradation, promoting stem elongation [28]. These results suggest that *XTHs* directly participate in the cell-wall-modification pathway and are regulated by plant hormone signals mediated by PBZ, such as Aux and GA, which influence plant growth and development.

Sucrose metabolism plays a pivotal role in development, mainly by generating a range of sugars as metabolites to fuel growth and essential compound synthesis. CWINs are key enzymes involved in sucrose metabolism and play important roles in plant growth and development. The CWIN-mediated release of hexose appears to facilitate sucrose uptake and cell division within filial tissues [31]. The *CWIN* family includes *CWIN1* (*Lin5*), *CWIN2* (*Lin6*), *CWIN3* (*Lin7*), and *CWIN4* (*Lin8*); *Lin5* is closely related to *Lin7*, and *Lin6* appears to be a paralogue of *Lin8*. *Lin7* may promote the division of cambium and cortical cells and increase the number of cell layers in *A. thaliana* stems by regulating the expression of cyclin D or cyclin-dependent kinase [32]. In the present study, *Lin5* and *Lin7* were upregulated by PBZ treatment, whereas *Lin6* and *Lin8* were downregulated (Figure 6D). This suggests that *Lin5* and *Lin7* may increase the number of cells, whereas *Lin6* and *Lin8* may reduce the cell volume. By converting sucrose into glucose and fructose, vacuolar invertases (VINs) double the osmotic contribution of sucrose, which has long been considered to play a major role in cell expansion owing to its osmotic effects and high VIN activity in many rapidly expanding tissues [33]. In *A. thaliana* roots, *AtVIN2* may regulate cell expansion through an osmosis-independent pathway [34]. In the present study, *VIN2* (*Lin9*) expression was downregulated by PBZ treatment (Figure 6D), indicating that *Lin9* may reduce the cell volume.

Flavonoids, a significant class of plant secondary metabolites crucial for growth and development, are biosynthesized via the phenylpropanoid pathway using phenylalanine from the shikimate pathway as a precursor [35,36]. This pathway converts phenylalanine to p-coumaroyl-CoA through the sequential actions of phenylalanine ammonia-lyase (PAL), cinnamic acid 4-hydroxylase (C4H), and 4-coumarate CoA ligase (4CL). PAL catalyzes the initiating step—the deamination of phenylalanine to trans-cinnamic acid—and plays a pivotal role in directing carbon flux from primary to secondary metabolism [37,38]. PAL and 4CL are frequently co-expressed [39]. Subsequently, chalcone synthase (CHS), a polyketide synthase, acts as the first rate-limiting enzyme dedicated to flavonoid formation [40,41]. Its functional importance is underscored by studies showing that CHS overexpression in *Arabidopsis* reduces Aux sensitivity and hypocotyl elongation [42,43], while its RNAi-mediated suppression in tomato decreases total flavonoid content [44]. In this study, *CHS1* was upregulated after 5 d of PBZ treatment, and then downregulated after 15 and 25 d. The expression of *CHS2* first increased, then decreased, reaching its lowest value at 15 d, and then rose again. These results indicate that *CHS1* did not affect the total flavonoid level, whereas *CHS2* might increase the flavonoid level in tomato epicotyls. Above all, although *SAURs*, *XTHs*, *Lin9* and *CHSs* may be involved in tomato epicotyl dwarfing, the specific molecular mechanisms require a more in-depth analysis.

## 4. Materials and Methods

### 4.1. Plant Materials and Treatments

Seeds of *Solanum lycopersicum* cv. DH tomato seedlings were subjected to surface sterilization using a 0.5% (*v/v*) sodium hypochlorite solution, followed by thorough rinsing with deionized distilled water. The seeds were then allowed to germinate in complete darkness at a constant temperature of 28 °C for 3 d. After germination, seedlings displaying uniform growth were carefully transferred to 72-hole trays filled with a growth medium composed of a mixture of peat, perlite, and vermiculite at a ratio of 3:1:1 (*v/v/v*). The seedlings were grown in a plant incubator during the day (12 h with 300 μmoL photons·m^−2^·s^−1^ illumination at 25 °C) and night (12 h at 15 °C). Throughout the experiment, seedlings were grown under consistent environmental conditions.

When the seedlings reached the two-leaf stage, 200 mg·L^−1^ PBZ (OriLeaf, Shanghai, China) (treatment), with a purity of 95%, and ddH_2_O (control) were sprayed on DH tomato plants. The distance from the sprayer to the tomato epicotyls was 5–6 cm, and the type of sprayer was a fine mist. In total, 20 plants were measured for epicotyl length and thickness at 0, 5, 15, and 25 d after PBZ spraying. Samples of the epicotyls were placed in liquid nitrogen for rapid freezing and were stored at –80 °C. The epicotyl samples of the PBZ treatment on different days were recorded as T5, T15, and T25, and those of the control on the corresponding days were recorded as C5, C15, and C25. The day of treatment was denoted as C0.

### 4.2. Microscopic Observation of Epicotyls

The epicotyls of the treatment and control groups at 0, 5, 15, and 25 d were subjected to microscopic observation. The paraffin sections were immersed in sequence in environmentally friendly dewaxing transparent liquid I and II for 20 min, respectively, added to anhydrous ethanol I and II for 5 min, respectively, and 75% ethyl alcohol for 5 min, and then rinsed with tap water. The tomato epicotyls slices were treated with toluidine blue for 2 min, rinsed with tap water, and dried in the oven at 60 °C. The slices were placed in xylene for 5 min and sealed with neutral gum. The sealed sections were placed under a microscope (Case Viewer 2.4, 3DHISTECH, Budapest, Hungary) to observe the cell numbers and sizes, and images were collected for analysis.

### 4.3. Plant Hormone Quantification

Endogenous GA1/3, GA4/7, IAA, and CTK contents were determined using an enzyme-linked immunosorbent assay produced at the Phytohormones Research Institute (China Agricultural University, Beijing, China) [45]. Plant hormones were extracted using 500 mL of an extract solution prepared by dissolving 1 mmol·L^−1^ butylated hydroxytoluene in 400 mL of 100% methanol, to which 100 mL of water was then added. Next, 1.0 g of tomato epicotyl tissue was added to 2 mL of the extract solution, with extraction at 4 °C for 4 h followed by centrifugation at 3500 r·min^−1^ for 8 min, and the supernatant passed through a C-18 solid-phase extraction column. An enzyme plate was used to determine plant hormone levels. The concentration of the standard substance and optical density of each sample at 490 nm were measured using an enzyme-linked immunospectrophotometer (Multiskan FC, Thermo Fisher Scientific, Waltham, MA, USA).

### 4.4. RNA Extraction and Transcriptome Analysis

To identify the key genes, we performed a high-throughput transcriptome analysis of tomato epicotyls sprayed with 200 mg·L^−1^ PBZ treatment after 5, 15, and 25 d. The epicotyls were then frozen in liquid nitrogen and stored. Total RNA was extracted using the MJZol Total RNA Extraction Kit (Majorbio, Shanghai, China). RNA concentration was measured using a Nanodrop2000 device (Thermo Fisher Scientific, Waltham, MA, USA). Agarose gel electrophoresis was used to assess RNA integrity, and an Agilent 5300 device (Agilent, Santa Clara, CA, USA) was used to determine the RQN value. All samples were subjected to quality checks. Three biological replicates were obtained for each genotype, and all samples were sequenced using the DNBSEQ-T7 platform. Reads were mapped to the Solanaceae Genomics Network (SGN ITAG release 4.0) [46], and transcript abundance was calculated as fragments per kilobase of transcript per million mapped reads. DEGs were determined by applying a threshold of an absolute fold change of ≥2.0 and a false discovery rate <0.05.

### 4.5. Reverse Transcription–Quantitative Polymerase Chain Reaction (RT–qPCR)

Total RNA was isolated from the epicotyls using a TaKaRa MiniBEST Universal RNA Extraction Kit (Takara, Beijing, China). The PrimeScript 1st strand cDNA Synthesis Kit (Tiangen, Beijing, China) was used to generate the first-strand cDNA. The cDNA was stored at −20 °C. RT-qPCR was conducted on a CFX Manager v. 3.1 Real-Time PCR System (Bio-Rad Laboratories, Hercules, CA, USA) using tomato *β-actin* as an internal reference [47]. Relative expression levels were calculated using the 2^−∆∆Ct^ method [48]. The mean of the three replicates was recorded. A melting curve analysis was performed to verify the specificity of the reaction. The primer sequences used for RT-qPCR are listed in Table S4.

### 4.6. Metabolomic Analysis

For the metabolomic analysis, metabolic profiling of materials is to be sampled simultaneously with the transcriptome (three biological replicates). A solid sample (100 mg) was added to a 2 mL centrifuge tube, and a 6 mm diameter grinding bead was added. Eight hundred microliters of extraction solution (methanol:water = 4:1 (*v:v*)) containing four internal standards (0.02 mg·mL^−1^ L-2-chlorophenylalanine, etc.) was used for metabolite extraction. Samples were ground using the Wonbio-96c (Shanghai Wanbo Biotechnology Co., Ltd., Shanghai, China) frozen tissue grinder for 6 min (−10 °C, 50 Hz), followed by low-temperature ultrasonic extraction for 30 min (5 °C, 40 kHz). The samples were left at −20 °C for 30 min, centrifuged for 15 min (4 °C, 13,000 *g*), and the supernatant was transferred to the injection vial for LC-MS/MS analysis. The LC-MS/MS analysis of the sample was conducted on a Thermo UHPLC-Q Exactive system at Majorbio Bio-Pharm Technology Co., Ltd. (Shanghai, China). An ACQUITY UPLC HSS T3 column (1.7 µm × 2.1 mm × 100 mm, Waters) was used for chromatographic separation. The LC conditions of HSS T3 columns were set as follows: the mobile phases consisted of 0.1% formic acid in water:acetonitrile (2:98, *v*/*v*) and 0.1% formic acid in acetonitrile. The flow rate was 0.40 mL·min^−1^ and the column temperature was 40 °C. The injection volume was 5 μL. The MS optimal conditions of HSS T3 columns were set as follows: source temperature = 400 °C; sheath gas flow rate = 40 arb; Aux gas flow rate = 10 arb; ion-spray voltage floating (ISVF) = −2800 V in negative mode and 3500 V in positive mode, respectively; and normalized collision energy = 20–40–60 V rolling for MS/MS. LC/MS raw data was pretreated using Progenesis QI 3.0 (Waters Corporation, Milford, MA, USA) software (Waters Corporation, Milford, MA, USA), and a three-dimensional data matrix in CSV format was exported. Metabolites were identified by searching the self-built plant-specific metabolite database of Majorbio Biotechnology Co., Ltd. (Shanghai, China). The data matrices obtained by searching the database in the two columns were merged, deduplicated, and uploaded to the MajorBio cloud platform (https://cloud.majorbio.com) for data analysis. Differential metabolites between the two groups were mapped to their biochemical pathways through metabolic enrichment and pathway analysis based on the KEGG database v3.0 (http://www.genome.jp/kegg/, accessed on 30 October 2025).

### 4.7. Statistical Analysis

Significant differences between the means of several treatments were determined using Duncan’s multiple range test af3ter running an ANOVA in SPSS (version 17.0; IBM Corp., Armonk, NY, USA). At least three biological replicates were used to calculate the standard deviation (±SD). Different letters indicate significant differences (*p* ≤ 0.05) according to Duncan’s test. The iPath pathway diagram was generated to visualize whether DEMs and DEGs were up-regulated or down-regulated. To further observe the changes and associations of metabolites and genes, the top 10 metabolites significantly different metabolites (VIP > 1, log_2_FC > 1, and *p* < 0.05) and significantly different genes (log_2_FC > 1, *p* < 0.05 and FPKM > 10) were used to describe a correlation network diagram. KEGG pathway was displayed using the MajorBio Biological Cloud platform (www.majorbio.com).

## 5. Conclusions

Both the flavonoid pathway and XTHs may block cell elongation in tomato epicotyls, resulting in dwarfism. Therefore, in future research, we aim to focus on the molecular mechanism by which flavonoid pathways, such as kaempferol and quercetin, in coordination with *XTH2* and *XTH3*, mediate Aux-responsive signaling to regulate tomato epicotyl dwarfism. In addition, *Lin9* in the sucrose metabolism pathway may serve as a candidate gene for epicotyl dwarfism in tomatoes.

## Data Availability

The raw sequence data of the transcriptome (GSA: CRA029889) and metabolome (OMIX011858) reported in this article have been deposited in the Genome Sequence Archive [49] in the National Genomics Data Center [50], China National Center for Bioinformation/Beijing Institute of Genomics, Chinese Academy of Sciences and are publicly accessible at https://ngdc.cncb.ac.cn/gsa, accessed on 30 October 2025.

## Supplementary Materials

The following supporting information can be downloaded at: https://www.mdpi.com/article/10.3390/plants14213311/s1, Table S1: Sequencing data statistics analyses of transcriptome. Table S2: TF family statistics analyses of transcriptome. Table S3: Differential metabolite statistical analyses of metabolome. Table S4: Primers used in this study.
