# Supplementary material for: Integrated Transcriptomic and Metabolomic Profiling of Paclobutrazol-Induced Dwarfism in Tomato Epicotyls
Source: Plants (Basel). 2025 Oct 30;14(21):3311. doi: 10.3390/plants14213311 (PMC12608322; doi:10.3390/plants14213311)
Supplement: Supplementary file 1 [file plants-14-03311-s001.zip › Table S1.pdf]

Table S1. Sequencing data statistics analyses of transcriptome

Sample	Clean reads	Clean bases	Q30(%)	GC content (%)
C0_1	43787078	6579617295	96.06	42.4
C0_2	44088440	6622573160	95.97	42.42
C0_3	44084358	6628045990	95.99	42.42
T5_1	44342556	6648818907	96.29	42.55
T5_2	41032234	6160025340	96.07	42.61
T5_3	43215272	6487341712	96.01	42.57
T15_1	45726372	6821815177	96.37	42.46
T15_2	42830406	6414741870	96.28	42.46
T15_3	42158462	6325448868	96.39	42.44
T25_1	43704230	6547347930	96.31	42.42
T25_2	51266568	7645760850	96.28	42.39
T25_3	41032364	6136541230	96.21	42.58
C5_1	43658076	6532262728	96.18	42.5
C5_2	43631784	6549936512	96.33	42.47
C5_3	42569584	6361002290	96.46	42.46
C15_1	42866850	6419877378	95.96	42.61
C15_2	40688274	6089568439	96.2	42.66
C15_3	46445036	6949885755	96.24	42.65
C25_1	40638604	6093918883	96.18	42.43
C25_2	40232874	6029376079	96.43	42.41
C25_3	45472090	6805421857	96.46	42.44

Note: Q30 is the percentage of bases with a clean reads mass value of ≥30 to evaluate the QC sequencing data.
