# Supplementary material for: Integrated Transcriptomic and Metabolomic Profiling of Paclobutrazol-Induced Dwarfism in Tomato Epicotyls
Source: Plants (Basel). 2025 Oct 30;14(21):3311. doi: 10.3390/plants14213311 (PMC12608322; doi:10.3390/plants14213311)
Supplement: Supplementary file 1 [file plants-14-03311-s001.zip › Table S2.pdf]

Table S2. TF family statistics analyses of transcriptome

TF family	Gene numbers	Transcript numbers
ERF	39	46
MYB	28	36
HB-other	24	36
NAC	23	35
WRKY	22	37
MYB_related	21	31
bHLH	20	31
B3	13	20
LBD(AS2/LOB)	11	14
bZIP	11	16
TCP	8	10
C2H2	6	7
M_type	6	8
NF-YA	6	20
C3H	5	7
GATA	5	7
GRAS	5	8
HSF	5	6
GRF	4	10
HD-ZIP	3	6
MIKC	3	4

YABBY	3	3
AP2	2	2
ARF	2	4
DBB	2	3
Dof	2	2
RAV	2	2
BBR-BPC	1	2
CO-like	1	1
E2F/DP	1	2
EIL	1	2
FAR1	1	1
SBP	1	2
SRS	1	1
TALE	1	3
ZF-HD	1	1

Note: TF family name: Name of the transcription factor family; Gene number: Number of genes belonging to this family; Transcript number: Number of transcripts belonging to this family.
