# Supplementary material for: Integrated Transcriptomic and Metabolomic Profiling of Paclobutrazol-Induced Dwarfism in Tomato Epicotyls
Source: Plants (Basel). 2025 Oct 30;14(21):3311. doi: 10.3390/plants14213311 (PMC12608322; doi:10.3390/plants14213311)
Supplement: Supplementary file 1 [file plants-14-03311-s001.zip › Table S3.pdf]

Table S3. Differential metabolites statistical analyses of metabolome

Differential group	Total differential number	Upregulation	Downregulation
C5 vs C0	144	109	35
C15 vs C0	209	144	65
C25 vs C0	219	152	67
T5 vs C0	168	120	48
T15 vs C0	185	165	20
T25 vs C0	211	179	32
C15 vs C5	174	105	69
C25 vs C5	176	115	61
C25 vs C15	182	98	84
T15 vs T5	179	158	21
T25 vs T5	200	161	39
T25 vs T15	192	144	48
T5 vs C5	134	69	65
T15 vs C15	173	123	50
T25 vs C25	186	148	38
Total	2732	1990	742
