# Supplementary material for: Integrated Transcriptomic and Metabolomic Profiling of Paclobutrazol-Induced Dwarfism in Tomato Epicotyls
Source: Plants (Basel). 2025 Oct 30;14(21):3311. doi: 10.3390/plants14213311 (PMC12608322; doi:10.3390/plants14213311)
Supplement: Supplementary file 1 [file plants-14-03311-s001.zip › Table S4.pdf]

Table S4. Primers used in this study

Gene ID	Gene name	Primers (5′-3′)
Solyc01g110910	*SAUR15A*	F: TATGGGCGAGTAAATGGG
		R: CACGCTAATCAAGAAACCG
Solyc01g110630	*SAUR9*	F: AACGATTCCCTGCTCAGAG
		R: ACTTTGTGGTGCTAAGACTCAC
Solyc03g123800	*MAPK2*	F: TAGGGAGTTCATTGCGTGC
		R: TAGTAGTAGCAGGACCCGTGTG
Solyc07g009380	*XTH2*	F: GACTGATTGGAGTAAAGCCC
		R: GCACCCATTTCATCCTTG
Solyc11g072310	*GA20ox2*	F: AGATAGTGTTGGTGGGCTTC
		R: TCTTCCGTTTGATAGTGCC
Solyc08g083110	*Solyc08g083110.4*	F: CAAGGGTCTCTTATGCTATTGG
		R: GCCTGGATACATCACTTTGAG
Solyc03g007890	*HSP17.6*	F: TCCTCCGTTACCACTCTACC
		R: AACTGCCTTCTTGCTCTCTC
Solyc09g010080	*Lin5*	F: AAAGGGATCTCAGCATCACAGG
		R: CGTCTTGGGCATATAGGTCAGC
Solyc10g083290	*Lin6*	F: ATCAAGCCCGATAACAATCCA
		R: CCTCACACTCCCAACCAATACTC
Solyc09g010090	*Lin7*	F: TTTGGTGCTGGTGGAAAGACA
		R: GGCTCCGTTCCGTTGTTAAAC
Solyc10g083300	*Lin8*	F: AAGGATGGGCGGGAATACA
		R: GGCCTGTGCTGGTGTGATT
Solyc08g079080	*Lin9*	F: ACTGGGTCAACCAACGAATC
		R: TGCCCTCATACTTGATCCAT
Solyc09g091510	*CHS1*	F: AGGAACTACTGGTGAAGGGC
		R: CCCACTAAGCAGCAACACTG
Solyc11g005330	*β-actin*	F: TGTCCCTATTTACGAGGGTTATGC
		R: AGTTAAATCACGACCAGCAAGAT
